# Incidence, risk factors, and prognosis of sciatic nerve injury in acetabular fractures: a retrospective cross-sectional study

**DOI:** 10.1007/s00264-024-06087-7

**Published:** 2024-01-09

**Authors:** Mahmood Arbash, Osama Z. Alzobi, Motasem Salameh, Mohd Alkhayarin, Ghalib Ahmed

**Affiliations:** 1 Department of Orthopedic Surgery, Surgical Specialty Center, Hamad Medical Corporation, Doha, Qatar; 2 Orthopaedics and Rehabilitation Department, Yale University School of Medicine, New Haven, CT USA

**Keywords:** Acetabular fractures, Injury, Prognosis, Sciatic nerve, Traumatic

## Abstract

**Purpose:**

This study aimed to investigate the incidence, risk factors of the sciatic nerve injury in patients with acetabulum fractures and assess its prognosis.

**Methods:**

A retrospective cross-sectional review was conducted on 273 patients with acetabulum fractures who were treated between January 1st, 2017, and December 30th, 2019. The medical records and radiographs of these patients were analyzed.

**Results:**

The overall nerve injury rate was 7.7% (21 of 273 cases), with 3.1% (8 of 273 cases) occurring because of the initial injury and 12.8% (13 of 101 cases) as post-operative complications. Among those with nerve injuries, 95.2% (20 of 21 cases) were males and the average age of the patients was 31.5 (SD 9.5) years. The most common mechanism of injury was motor vehicle collisions with 55.7% (152 of 273 cases), and the most common fracture pattern associated with nerve injury was posterior column and posterior wall fracture with 31.6% (6 of 21 cases). Hip dislocation was found in 16.5% (14 of 21 cases) of patients with nerve injury. The Kocher Langenbeck approach was the most common approach used for patients with post-operative nerve injury, and the prone position was significantly associated with sciatic nerve injury during the operation. Of all patients with nerve injury, 52% (11 of 21 cases) had fully recovered, 29% (6 of 21 cases) had partially recovered, and 19% (4 of 21 cases) had no improvement. The average follow-up was 15 months.

**Conclusion:**

This study emphasizes the incidence of sciatic nerve injuries in individuals with acetabulum fractures and highlights key risk factors, including hip dislocation, posterior column, and posterior wall fractures. It is noteworthy that the Kocher Langenbeck approach and the prone position may contribute to iatrogenic nerve injuries. Encouragingly, over half of the patients who suffered nerve injuries achieved full recovery, while nearly one-third experienced partial recovery. These findings underscore the vital significance of recognizing and addressing these risk factors in clinical practice.

## Introduction

Acetabular fractures are common injuries that associated with high-energy trauma, such as a road traffic accident or a fall from a significant height [1, 2]. These fractures are typically associated with other orthopaedic or non-orthopaedic injuries, especially in younger patients, with rates ranging from 40 to 75% [3]. Displaced acetabular fractures may experience significant pain and disability, necessitating surgical intervention to fix the affected bone [4].

Sciatic nerve injury is one of the potential complications of acetabular fractures which can occur either because of the initial trauma or during the surgical reconstruction (iatrogenic) and can cause significant morbidity, including motor and sensory deficits, chronic pain, and loss of function. Previous studies have reported the incidence of sciatic nerve injury between 5–33% in patients with various types of acetabulum fractures [5]. A recent meta-analysis found that the incidence of post-traumatic and iatrogenic sciatic nerve injury associated with acetabular fractures was 9% and 5%, respectively [6].

The development of sciatic nerve injury is influenced by various factors, including initial trauma and patient-related factors, such as obesity [5]. Post-operative nerve injury is often associated with haematoma and heterotopic ossification [7]. The Kocher-Langenbeck approach and prone position for fixing acetabulum fractures was associated with a higher incidence of intervention-related nerve injuries [8, 9]. This is especially true when a fracture pattern involves the posterior wall or posterior column [5, 6, 9].

A comprehensive understanding of the factors contributing to sciatic nerve injury in patients with acetabular fractures is essential as it assists in identifying patients at higher risk for this complication, guides surgeons in selecting appropriate treatment options, and provides valuable insights into the patient's prognosis and neurological recovery [5, 6].

While several studies have addressed the incidence and potential risk factors of nerve injury in patients with acetabular fractures, there is limited data available on the recovery of neurological function following such injuries [7, 8]. This study aimed to assess both the incidence and potential risk factors contributing to nerve injury in patients with acetabular fractures and underwent operative or non-operative treatment, and to evaluate the subsequent prognosis of nerve injury, an aspect that holds particular importance and is under represented in existing literature.

## Materials and methods

### Data collection

The fracture registry of a single level I trauma centre was utilized to identify electronic medical records of all acetabulum fracture patients who were admitted and treated in the hospital between January 1st, 2017, and December 30th, 2019. The Institutional Review Board (MRC-01–20-538) approved the study. The sample size was not calculated for this study because all patients with acetabulum fractures during the study period and met the inclusion criteria was included in this study, given the retrospective, cross-sectional nature of the study [10].

All acetabulum fractures in skeletally mature patients (age > 18 years) with complete medical records during the study period were included. Patients with severe traumatic brain injury or spinal cord injury that hindered proper neurological examination were excluded from the study. Demographic data, including age, sex, and body mass index, were collected from the electronic medical records in our institute. The mechanism of injury, associated orthopaedic and non-orthopaedic injuries, associated hip dislocation, fracture pattern according to the Letournel classification system using plain radiography and computerized tomography scans, sciatic nerve injury at admission with its associated motor and sensory deficit, the surgical approach utilized in the operated cases, position of the patient during the operation. Intraoperative physiological nerve monitoring was not performed. The study identified the injured nerves and described the motor and sensory deficits associated with them. The timing and extent of nerve recovery were also documented, including cases of no recovery, partial recovery, or complete recovery. Data were retrieved by trained personnel not involved in patient care. All operative cases were performed by two experienced orthopaedic trauma surgeons with considerable experience in treating acetabulum fractures.

### Statistical analysis

Statistical analysis was performed using SPSS 28.1.1. Categorical variables were expressed as absolute numbers and percentages. The Pearson chi-square test was used for the association between two variables. Continuous data were expressed as mean, median, and standard deviation. The Wilcoxon rank-sum test was used for the comparison of continuous variables. The study set the alpha value at 0.05 throughout the analysis.

## Result

### Demographic characteristics

A total of 273 (245 males, 28 females) patients admitted to our institution were included in the study. The mean age was 34.6 years (range 18 –84), with a male predominance in 89.7% (245 of 273 cases). The average body mass index (BMI) was 26.5 ± 5.9 kg/m2 (The demographic characteristics were summarized in Table 1).

**Table 1:** Demographics and injury characteristics for patients with and without nerve injury

Patients’ numbers	All patients (273)	Sciatic nerve injury (21, 7.7%)	No nerve injury (252, 92.3%)
Age (SD)	34.6 (11.6)	31.5 (9.5)	34.85 (11.8)
Males	245 (89.7%)	20 (8.2%)	225 (91.8%)
BMI	26.49 ± 5.9	27.86 ± 5.9	26.4 ± 5.9
Mechanism of injury			
MVC	152 (55.7%)	15 (9.9%)	137 (90.1%)
FFH	67 (24.5%)	1 (1.5%)	66 (98.5%)
Fall of heavy object	17 (6.2%)	2 (11.8%)	15 (88.2%)
Pedestrian	26 (9.5%)	3 (11.5%)	23 (88.5%)
ATV	6 (2.2%)	0 (0%)	6 (100%)
Assault	1 (0.4%)	0 (0%)	1 (100%)
Cyclist	4 (1.5%)	0 (0%)	4 (100%)
Polytrauma	156 (57.1%)	11 (7.1%)	145 (92.9%)
Isolated injury	117 (42.9%)	10 (8.5%)	107 (91.5%)
Associated Hip dislocation	85 (31.1%)	14 (16.5%)	71 (83.5%)
Surgical management	101 (37%)		
Iatrogenic sciatic nerve injury		13 (12.87%)	
Post-traumatic injury		8 (3.1%)	
Conservative management	172 (63%)		
Classifications			
Anterior column fracture	66 (24.2%)	0 (0%)	66 (100%)
Anterior wall fracture	27 (9.9%)	0 (0%)	27 (100%)
Posterior column fracture	21 (7.7%)	2 (9.5%)	19 (90.5%)
Posterior wall fracture	69 (25.3%)	4 (5.8%)	65 (94.2%)
Transverse fracture	8 (2.9%)	1 (12.5%)	7 (87.5%)
T-type fracture	8 (2.9%)	2 (25%)	6 (75%)
Transverse and posterior wall fracture	17 (6.2%)	4 (23.5%)	13 (76.5%)
Posterior column & posterior wall fracture	19 (7.0%)	6 (31.6%)	13 (68.4%)
Anterior column and posterior hemi-transverse	14 (5.1%)	1 (7.1%)	13 (92.9%)
Associated both columns fracture	21 (7.7%)	1 (4.8%)	20 (95.2%)
Not classifiable fracture	3 (1.1%)	0 (0%)	3 (100%)

^*^SD Standard deviation, *BMI* Body mass index, *MVC* Motor vehicle collision, *FFH* Fall from height, *ATV* All-terrain vehicle

### Mechanism of injury

**Fig. 1:**
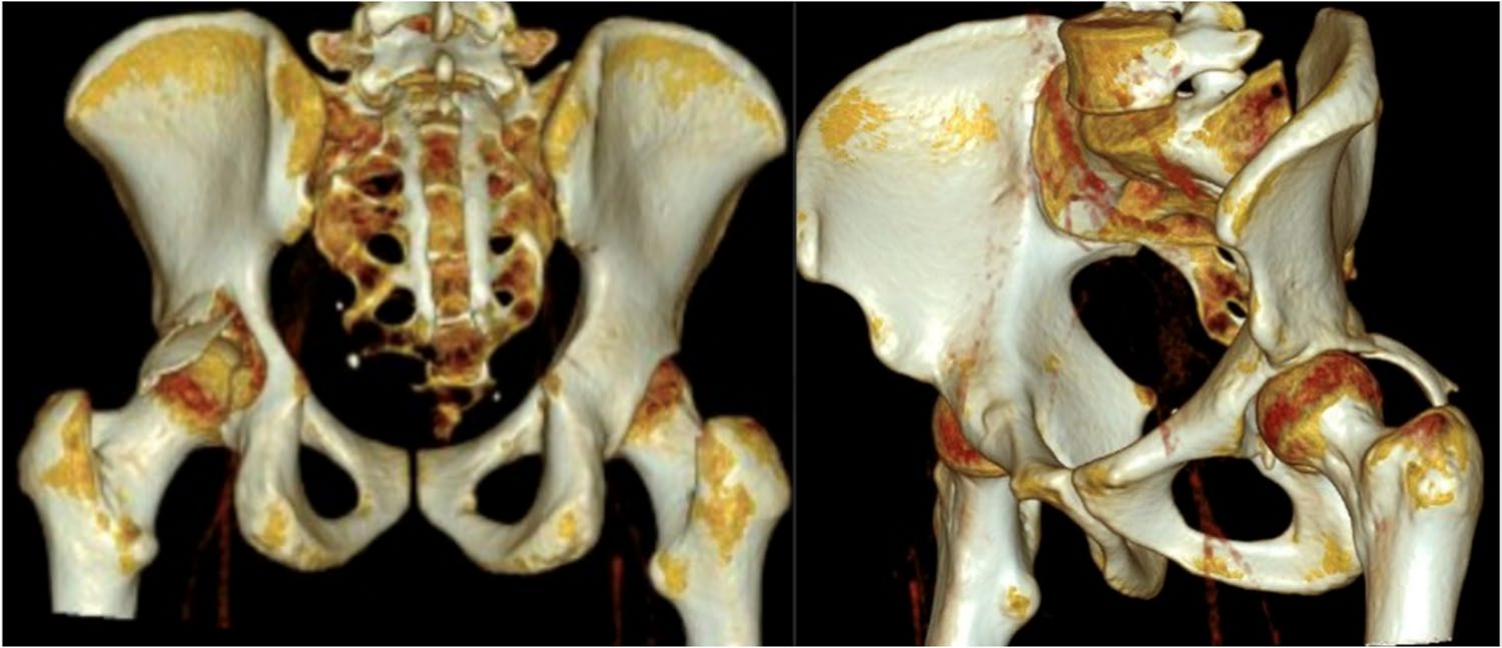
Three-dimensional CT scan for a patient with posterior wall fracture

The leading cause of injury in the studied group was motor vehicle collisions (MVC), which accounted for 55.7% (152 of 273 cases), followed by falls from heights (FFH) at 24.5% (67 of 273 cases), pedestrians hit by cars at 9.5% (26 of 273 cases), falls of heavy objects at 6.2% (17 of 273 cases), All-terrain vehicle (ATV) rollovers at 2.2% (6 of 273 cases), cyclists hit by cars at 1.5% (4 of 273 cases), and lastly, assault at 0.4% (1 of 273 cases) (Fig. 1).

### Types of fracture, treatment provided and associated injuries.

Based on the Letournel’s classification system, the most frequent fracture pattern was the posterior wall fracture in 25.3% (69 of 273 cases), followed by the anterior column fracture in 24% (66 of 273 cases). Other fracture patterns included anterior wall fracture in 9.9% (27 of 273 cases), posterior column fracture in 7.7% (21 of 273 cases), associated both column fracture in 7.7% (21 of 273 cases), associated posterior column and posterior wall fracture in 7% (19 of 273 cases), associated transverse fracture and posterior wall fracture in 6.2% (17 of 273 cases), associated anterior column and posterior hemi-transverse fracture in 5.1% (14 of 273 cases), transverse fracture in 2.9% (8 of 273 cases), T-type fracture in 2.9% (8 of 273 cases), while 1.1% of cases (3 of 273 case) were not classified.

Surgical fixation of acetabulum fractures was performed in 101 out of 273 patients and 172 were treated with conservative management. The average time from injury to surgery was 5.5 days. The Kocher-Langenbeck’s approach was the most utilized surgical method, being employed in 76.2% (77 out of 101 patients) of the cases. Other approaches implemented included the ilioinguinal approach in 6.9% (7 out of 101 cases), the modified Stoppa’s approach in 9.9% (10 out of 101 cases), and percutaneous screw fixation in 6.9% (7 out of 101 cases).

Three positioning during surgery were observed: lateral in 58% (58 of 101 cases), supine in 22.8% (23 of 101 cases), and prone in 19.8% (20 of 101 cases). According to our study, hip dislocation was present in 85 cases (31.1%). Additionally, 156 patients (57.1%) experienced polytrauma injuries. Overall, three cases were complicated by wound infection.

### Incidence of sciatic nerve injury

**Fig. 2:**
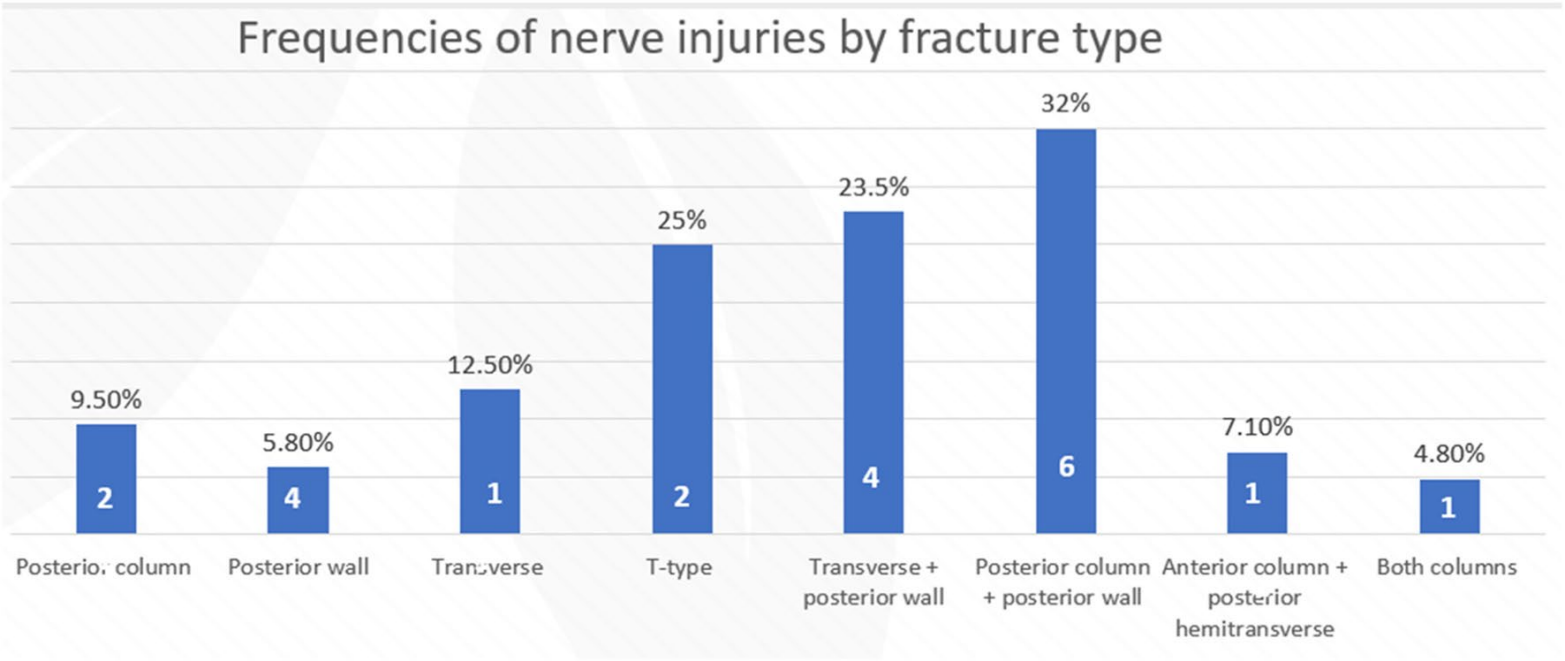
The distribution of patients categorized by fracture classification

The overall nerve injury rate was 7.7% (21 of 273 cases), with 3.1% (8 of 273 cases) occurring because of the initial injury and 12.9% (13 of 101 cases) as iatrogenic post-operative on complications (Fig. 2).

Among those with nerve injuries, 95.2% (20 of 21 patients) were males. The average age of the patients with nerve injuries was 31.5 (SD 9.5) years, and the average body mass index was 27.86 ± 5.9 kg/m2. Out of all the patients with sciatic nerve injuries, 85.7% (18 of 21 cases) experienced a common peroneal nerve injury, and 14.3% (3 of 21 cases) suffered from a complete sciatic nerve injury (Table 2). Out of the patients who suffered from iatrogenic sciatic nerve injury, 12 underwent open reduction and internal fixation of acetabulum fractures using the Kocher-Langenbeck approach, and one was operated through the ilioinguinal approach. The sciatic nerve was observed intraoperatively during the procedure, and no apparent abnormalities were detected. None of the patients received neurolysis as part of their treatment. At the follow-up period, nearly 80% of the patients exhibited either complete or partial recovery.

**Table 2:** Types of nerve injuries are detailed based on the amount of recovery: None, partial, or complete nerve recovery

	All sciatic nerve injuries (21, 100%)	No recovery (4, 19%)	No recovery (4, 19%)	Complete recovery (11, 52.4%)
Length of clinical follow-up (mean, months)	15			
Recovery time (mean, months)	9		12	5.7
Injury mechanism				
Traumatic	8	1	2	5
Iatrogenic	13	3	4	6
Nerve injured				
Sciatic complete injury	3	0	1	2
Sciatic with CPN only	18	4	5	9
Tibial nerve only	0			
Surgical approach				
Ilioinguinal	1	0	1	0
Kocher–Langenbeck	12	3	3	6
Surgical position				
Lateral	3	0	0	3
Prone	9	3	3	3
Supine	1	0	1	0
Associated Hip dislocation	14	3	4	7

*CPN* Common peroneal nerve

### Risk factors for sciatic nerve injury

The study analyzed the potential association between various factors and nerve injury in patients with acetabulum fractures. The most common mode of injury was MVC (15 cases), followed by a pedestrian hit by a car (3 cases), fall of heavy object (2 cases), and fall from height (FFH) (1 case). In patients experiencing post-traumatic nerve injury, comprising 8 cases, there was a significant association with fractures involving both the posterior wall and column, noted in 4 cases (*p* < 0.01). Subsequent fracture patterns included one case of transverse fracture with posterior wall fracture, one case of a transverse fracture, one case of both column fractures, and one case of a T-shaped fracture. In patients with iatrogenic nerve injury in 13 cases, the fracture patterns were observed as follows: four cases exhibited fractures of the posterior wall, three cases featured both posterior wall and column fractures, and another three cases presented associated transverse and posterior wall fractures. Additionally, there was one case each of transverse fracture, posterior column fracture, and T-shaped fracture. Hip dislocation was found in 14 patients with nerve injury (66.6%), with a statistical difference between the group of patients with intact nerve and the group of patients with nerve injury (*p* < 0.001). Nine events were reported with iatrogenic group and 5 associated with post-traumatic nerve injury. The most common approach used for patients with iatrogenic nerve injury surgery was the Kocher-Langenbeck approach (*n* = 12 out of 13 cases). The prone position was the most common position during surgery for patients with iatrogenic nerve injury (9 cases), followed by the lateral position (3 cases) and the supine position (1 case). Iatrogenic sciatic nerve injury was significantly associated with a prone position during the operation (*p* < 0.001).

### Prognosis of sciatic nerve injury

**Fig. 3:**
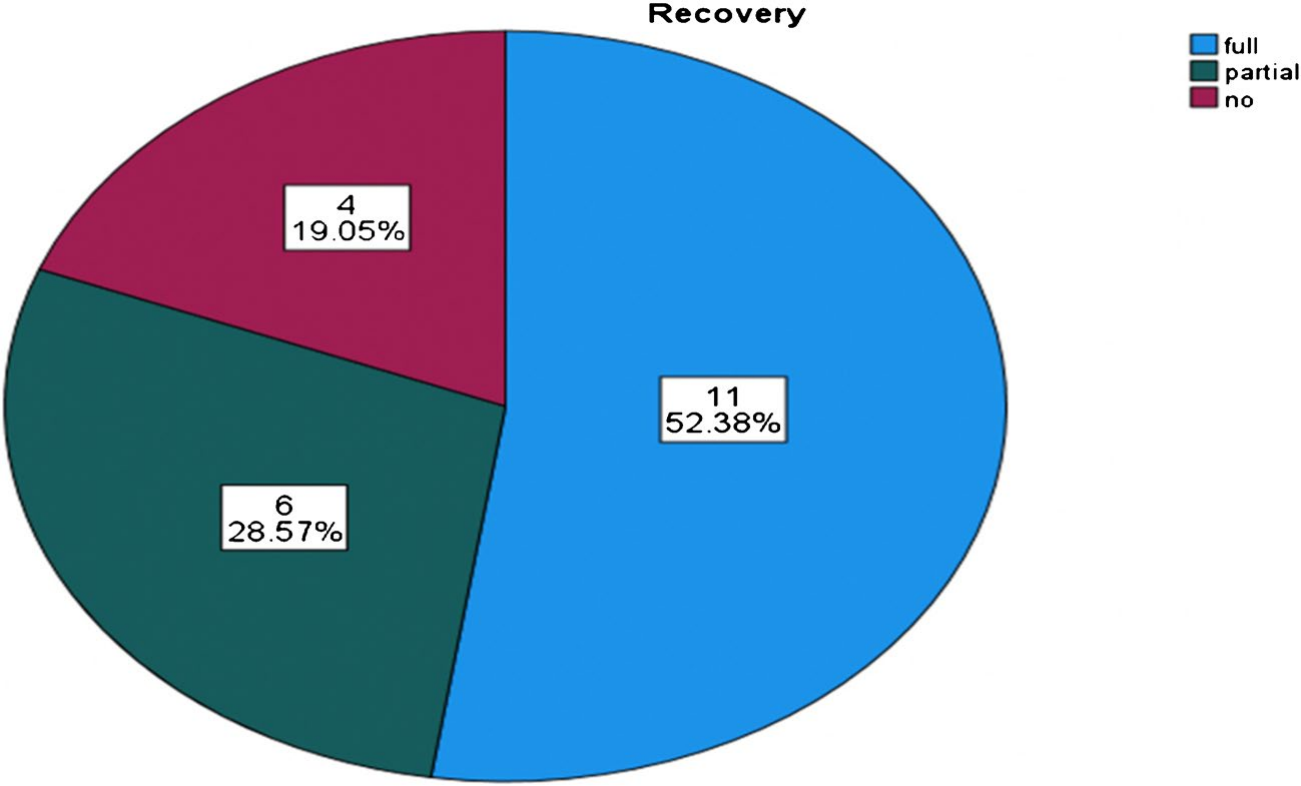
The distribution of patients with sciatic nerve injury according to nerve recovery

Out of the 21 patients experiencing nerve injury, 52.38% (11 of 21) achieved full recovery, 28.57% (6 of 21) exhibited partial recovery, and 19.05% (4 of 21) showed no improvement (Fig. 3).

The mean time for partial nerve recovery was 12 months and for complete nerve recovery was 5.6 months (Table 2). Among those who displayed no progress, three had a posterior dislocation, and three incurred an iatrogenic sciatic nerve injury during surgery while in the prone position using the posterior approach. The median follow-up time was notably longer for patients without nerve recovery, standing at 17 months (Table 2). Nerve conduction studies were conducted only for two patients, revealing complete common peroneal dysfunction in both cases.

## Discussion

The most significant finding of this study is that the overall incidence of sciatic nerve palsy in patients with acetabulum fractures was 7.7%. Of this percentage, 3.1% resulted from the initial injury. Moreover, 12.87% of surgically treated patients experienced post-operative complications resulting in sciatic nerve palsy. Previous literature reported a wide variation in documented incidence of post-traumatic and iatrogenic sciatic nerve palsy, which ranged from 3 to 30% [6, 11–14].

In their meta-analysis, Stavrakakis et al. noted a 5.1% rate of post-traumatic sciatic nerve injuries and a 1.4% rate for iatrogenic injuries [11]. Similarly, Hakeem et al. found rates of 9% for post-traumatic and 5% for iatrogenic sciatic nerve injuries related to acetabular fractures [6]. Our findings align with the reported rates of post-traumatic sciatic nerve injuries in these studies, but show a slightly higher incidence for iatrogenic injuries.

Acetabular fractures are typically the result of high-energy mechanisms. Our investigation for potential risk factors revealed that the most prevalent mode of injury was MVC, which accounted for 71.4% of sciatic nerve injuries. This finding aligns with the results of previous studies, including Simske et al., who reported that 59.4% of sciatic nerve injuries were caused by motor vehicle accidents [5]. Additionally, our study demonstrated a statistically significant correlation between nerve injury and posterior column and posterior wall fractures (*p* < 0.001). This is consistent with König et al., who reported posterior column and posterior wall fractures as the most common fracture patterns associated with sciatic nerve injury [15]. The initial mechanism of injury might not have a direct relationship with iatrogenic injury. However, it could indirectly influence iatrogenic injury by affecting the type and location of the fracture, which in turn influences the surgical approach and technique [16].

Acetabular fractures, especially when accompanied by dislocation, are linked to poorer long-term functional outcomes and a higher risk of complications [17]. In particular, the incidence of sciatic nerve injury was significantly associated with acetabular fracture and traumatic posterior dislocation of the hip, with some studies reporting occurrences as high as 47% [18]. Our study revealed a statistically significant association between posterior hip dislocation and nerve injury, with 66.6% of the patients with nerve injury having a posterior hip dislocation (*p* < 0.001).

In addition to traumatic causes, sciatic nerve injuries can also occur iatrogenically, and our study found that patient position and surgical approach were significant risk factors. The prone position was identified as a significant risk factor for sciatic nerve injury, with 9 out of 13 cases occurring in this position. This finding is consistent with a study by Salameh et al. [9], who reported a significantly higher incidence of iatrogenic sciatic nerve injury in the prone group. The authors acknowledged that nerve injury might be due to the prolonged use of retractors required for exposure in the prone position and the longer total operative time in this group. Moreover, due to the high incidence of posterior-based injuries such as posterior wall, posterior column, and posterior hip fracture-dislocation, the posterior surgical approach is frequently utilized [19, 20]. Among patients who had sciatic nerve injury, the most frequently used approach was the Kocher Langenbeck approach (12 out of 13 cases). Nonetheless, sciatic nerve injuries can also occur with other approaches. For instance, Simske et al. found that all eight iatrogenic nerve injuries occurred during the use of the ilioinguinal approach [5].

Previous reports in the literature indicated that there was a significant amount of spontaneous recovery of sciatic nerve injuries with favourable outcomes and can occur in around 70% of cases, although some cases lead to permanent disability [11, 21]. Treatment options include ankle–foot orthosis for temporary nerve lesions or tendon transfers for permanent disability [22, 23]. In another review, recovery rates for iatrogenic and post-traumatic sciatic nerve injury associated with acetabular fractures were reported to be 55% and 68%, respectively [4]. Our study found that 19% of nerve injury patients did not experience any recovery, while 28.6% experienced partial recovery and 52.4% achieved complete recovery.

This study suggests several measures to mitigate iatrogenic sciatic nerve injury in acetabulum fractures. Careful consideration of surgical positioning is essential, given the higher incidence of injuries in the prone position. A thoughtful choice and execution of the surgical approach, tailored to the specific fracture type and location, are also crucial, with particular attention to approaches previously associated with higher injury rates, such as the Kocher-Langenbeck and ilioinguinal approaches. Addressing associated conditions like posterior hip dislocation promptly and effectively is additionally vital. Further research is needed to explore and validate these and additional preventive strategies, recognizing the limitations of our retrospective study.

Owing to the retrospective nature of this study, several limitations must be acknowledged, including the inevitable selection bias. Other limitations were the inclusion of the only adult population, and all the cases were not operated by the same surgeon. Additionally, we acknowledge that the restriction to a three-year timeframe for case recruitment served as a limiting factor in this study. Finally, our follow-up protocol did not cover factors such as rehabilitation and physiotherapy, which may affect the results. Furthermore, a longer follow-up would have enabled us to better observe the long-term outcomes and nerve recovery.

## Conclusion

In summary, this study emphasizes the incidence of sciatic nerve injuries in individuals with acetabulum fractures and highlights key risk factors, including hip dislocation, posterior column, and posterior wall fractures. It is noteworthy that the Kocher Langenbeck approach and the prone position may contribute to iatrogenic nerve injuries. Encouragingly, over half of the patients who suffered nerve injuries achieved full recovery, while nearly one-third experienced partial recovery. These findings underscore the vital significance of recognizing and addressing these risk factors in clinical practice.

## Data Availability

Happy to provide access to data (coding) upon request.
